# Pre and postoperative lactate levels and lactate clearance in predicting in-hospital mortality after surgery for gastrointestinal perforation

**DOI:** 10.1186/s12893-022-01479-1

**Published:** 2022-03-09

**Authors:** Min Kyu Kang, Seung-Young Oh, Hannah Lee, Ho Geol Ryu

**Affiliations:** 1 Department of Surgery, Seoul National University College of Medicine, Seoul, Korea; 2 Department of Critical Care Medicine , Seoul National University Hospital, Seoul, Korea; 3 Department of Anesthesiology, Seoul National University College of Medicine, Seoul, Korea

**Keywords:** Gastrointestinal perforation, In-hospital mortality, Risk factor, Perioperative lactate

## Abstract

**Background:**

This study aimed to compare the prognostic significance of pre and postoperative lactate levels and postoperative lactate clearance in the prediction of in-hospital mortality after surgery for gastrointestinal (GI) perforation.

**Methods:**

Among patients who underwent surgery for GI perforation between 2013 and 2017, only patients whose lactate were measured before and after surgery were included and divided into an in-hospital mortality group and a survival group. Data on demographics, comorbidities, pre and postoperative laboratory test results, and operative findings were collected. Risk factors for in-hospital mortality were identified, and receiver-operating characteristic (ROC) curve analysis was performed for pre and postoperative lactate levels and postoperative lactate clearance.

**Results:**

Of 104 included patients, 17 patients (16.3%) died before discharge. The in-hospital mortality group demonstrated higher preoperative lactate (6.3 ± 5.1 vs. 3.5 ± 3.2, *P* = 0.013), SOFA score (4.5 ± 1.7 vs. 3.4 ± 2.3, P = 0.004), proportions of patients with lymphoma (23.5% vs. 2.3%, *P* = 0.006), and rates of contaminated ascites (94.1% vs. 68.2%, *P* = 0.036) and lower preoperative hemoglobin (10.4 ± 1.6 vs. 11.8 ± 2.4, *P* = 0.018) compare to the survival group. Multivariate analysis revealed that postoperative lactate (HR 1.259, 95% CI 1.084–1.463, *P* = 0.003) and preoperative hemoglobin (HR 0.707, 95% CI 0.520–0.959, *P* = 0.026) affected in-hospital mortality. In the ROC curve analysis, the largest area under the curve (AUC) was shown in the postoperative lactate level (AUC = 0.771, 95% CI 0.678–0.848).

**Conclusion:**

Of perioperative lactate levels in patients underwent surgery for GI perforation, postoperative lactate was the strongest predictor for in-hospital mortality.

**Supplementary Information:**

The online version contains supplementary material available at 10.1186/s12893-022-01479-1.

## Introduction

Gastrointestinal (GI) perforation is one of the most common indications of emergency surgery performed in surgical departments and poses a high risk of mortality for surgical patients, with a mortality rate of 15–33% in critically ill patients suffering from peritonitis [1–4]. Since the mortality rate significantly varies depending on the timing of intervention, age, and underlying comorbidities [5], composite indexes such as the Mannheim Prognostic Index (MPI) and Physiological and Operative Severity Score for enUmeration of Mortality and morbidity (POSSUM) score are widely used to stratify patients into risk groups and predict risk-adjusted mortality [3, 6]. Although these scores include several factors, some of them need to be modified and new factors need to be included to suit a modern patient. Indeed, some studies have suggested that the age cut-off, which is > 50 years in MPI, should be increased [7].

Lactate, although not included in the composite indexes, is a powerful single factor known to predict the prognosis in various situations. In the emergency department patients, serum lactate levels above 4.0 mmol/L have been reported to be associated with poor prognosis regardless of infection [8]. In the out-of-hospital cardiac arrest patients, serum lactate levels at the time of admission were higher in patients with an unfavorable outcomes [9, 10]. In the patients with sepsis, serum lactate levels above 1.9 mmol/L was one of the strongest predictor for intensive care unit admission [11]. Particularly with the development of the Surviving Sepsis Campaign [12], lactate began to attract attention as a prognostic marker in infected patients including patients with peritonitis.

In the case of studies that assessed the role of lactate purely in surgical patients, the timing of lactate level measurement was not constant; some focused on preoperative lactate levels and others focused on postoperative lactate levels [2, 5, 13]. However, the preoperative or postoperative lactate level alone does not reflect the effect of surgery to completely eliminate the cause of septic shock or the patient’s ability to metabolize the produced lactate. Lactate clearance has been known as a potential predictor for ICU admission in patients with sepsis [11]. And in particular, it has been reported that low lactate clearance is associated with high in-hospital mortality in patients with sepsis with non-pneumonia cause [14]. Peritonitis is one of the non-pneumonic cause, and the surgery can increase the rate of lactate elimination through massive resuscitation during surgery and decrease the rate of lactate production through septic cause removal. For these reasons, lactate clearance, which reflects changes with time of production and elimination of lactate, rather than single level at certain time point, may be more appropriate for predicting the prognosis in sepsis patients underwent surgery.

This study aimed to evaluate the prognostic significance of perioperative lactate levels such as pre and postoperative lactate levels and postoperative lactate clearance in the prediction of in-hospital mortality in patients who underwent surgery for GI perforation.

## Methods

This study was a retrospective cohort study whose protocol was approved by the institutional review board of Seoul National University Hospital (SNUH) (No. 1711-109-901). Informed consent was waived due to the retrospective study design. All procedures followed were in accordance with the ethical standards of the responsible committee on human experimentation and with the Helsinki Declaration of 1964 and later versions and adhered to the relevant guidelines.

Among adult patients (age ≥ 18 years) who admitted intensive care unit after surgery for GI perforation between 2013 and 2017 at SNUH, a tertiary center in South Korea, only patients whose lactate levels were measured before and after surgery were included in this study. Patients with secondary iatrogenic GI perforation from other surgeries and those who underwent surgery for GI perforation in other surgical departments and divisions were excluded.

Data collection was carried out by a review of electronic medical records. For the evaluation of risk factors for in-hospital mortality in surgical patients with GI perforation, data on preoperative variables, operation-related variables, and postoperative variables were collected. To adjust for the patients’ comorbidities which could affect mortality, the Charlson Comorbidity Index (CCI) score derived from 20 diseases was used [15]. Preoperative variables included white blood cell (WBC) count, hemoglobin level, albumin level, C-reactive protein (CRP) level, lactate level, the Sequential Organ Failure Assessment (SOFA) score, and time to surgery from diagnosis. Operation-related variables such as the type of surgery, perforation site, the cause of perforation and characteristics of ascites observed during surgery were obtained. The balance of fluid input and output during surgery was included to reflect the degree of intraoperative resuscitation. Postoperative variables included the lactate level immediate after surgery, postoperative lactate clearance, and perforation-related re-operation rate. The lactate clearance was calculated according to the following equation [16, 17].

$${\text{Postoperative}}\,{\text{lactate }}\,{\text{clearance }}\left( {\text{\% }} \right)\, = \,{ }\frac{{{\text{Lactate }}\,{\text{preoperative}}\, - \,{\text{Lactate }}\,{\text{postoperative}}}}{{{\text{Lactate }}\,{\text{preoperative}}}} \times 100$$

Analysis for normal distribution was performed for all variables, and an appropriate comparison method was selected for each variable. The characteristics and potential risk factors of patients who died before discharge and those who survived were compared using the Chi-square and Fisher's exact tests for categorical variables, and Student’s t-test and Mann–Whitney U-test were used for continuous variables, respectively. A multivariate analysis using a logistic regression with backwards stepwise regression method was performed on factors that were significantly associated with in-hospital mortality in the univariate analysis. The model fitness was assessed by the Hosmer–Lemeshow test and Nagelkerke R2 were used to evaluate the model. A *P*-value < 0.05 was considered indicative of statistical significance. To evaluate the predictive values of pre and postoperative lactate levels and postoperative lactate clearance, a receiver operating characteristic (ROC) curve analysis was performed. We calculated the optimal cut-off points of each of them based on Youden index and also calculated sensitivity and specificity. And area under the curve (AUC) for pre and postoperative lactate levels and postoperative lactate clearance were compared with each other. Statistical analyses were performed using IBM SPSS Statistics for Windows, Version 22.0 (IBM Corp., Armonk, NY).

## Results

**Fig. 1:**
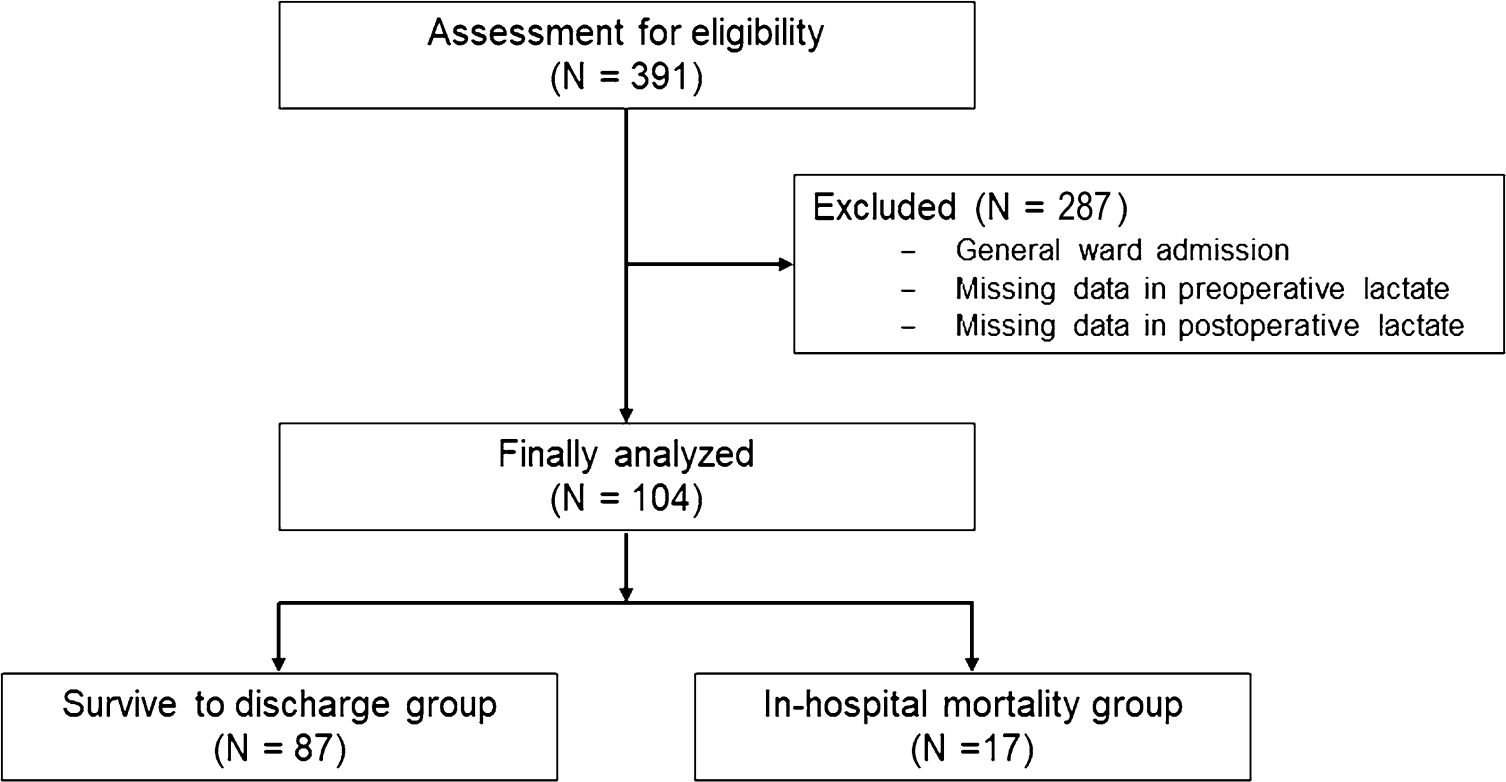
Flow chart

Among 391 patients who underwent surgery for GI perforation, 104 patients were included and 17 patients (16.2%) died before discharge (Fig. 1). Patients who died before discharge were classified in the in-hospital mortality group, and patients who survived were classified into the survival group. No significant differences were observed between the two groups regarding patient characteristics (Table 1).

**Table 1:** Patient characteristics

Characteristics	Survive to discharge (N = 87)	In-hospital mortality (N = 17)	*P*-value
Age (years)	65.3 ± 14.8	67.3 ± 12.4	0.602
Male/female	46:41	7:10	0.378
Body mass index (kg/m^2^)	21.8 ± 3.8	21.7 ± 3.7	0.927
Comorbidities			
Hypertension	46 (52.9)	6 (35.3)	0.185
Diabetes mellitus	19 (21.8)	5 (29.4)	0.498
Chronic liver disease	11 (12.6)	3 (17.6)	0.697*
Chronic kidney disease	9 (10.3)	4 (23.5)	0.220*
Coronary artery disease	10 (11.5)	3 (17.6)	0.442*
Cerebrovascular disease	4 (4.6)	1 (5.9)	1.000*
Charlson Comorbidity Index score	4.7 ± 2.7	5.8 ± 2.6	0.097

Data are presented as mean ± SD, or number (%)

*Fisher’s exact test

In the univariate analysis, the patients in the mortality group demonstrated significantly higher preoperative lactate levels (6.3 ± 5.1 vs. 3.5 ± 3.2, *P* = 0.013) and SOFA score (4.5 ± 1.7 vs. 3.7 ± 3.2, *P* = 0.004). Preoperative hemoglobin levels (10.4 ± 1.6 vs. 11.8 ± 2.4, *P* = 0.018) and albumin levels (2.5 ± 0.7 vs. 3.0 ± 0.7, *P* = 0.017) were significantly lower in the in-hospital mortality group than the survival group (Table 2). Regarding intraoperative and postoperative variables, the proportion of contaminated ascites (94.1% vs. 68.2%, *P* = 0.036) and postoperative lactate level (7.9 ± 4.9 vs. 3.6 ± 3.0, *P* < 0.001) were higher in the in-hospital mortality group than the survival group (Tables 3 and 4).

**Table 2:** Preoperative factors

Characteristics	Survive to discharge (N = 87)	In-hospital mortality (N = 17)	*P*-value
WBC count (n/μL)	10,409 ± 9795	9305 ± 6439	0.657
Hemoglobin (g/dL)	11.8 ± 2.4	10.4 ± 1.6	0.018
Albumin (g/dL)	3.0 ± 0.7	2.5 ± 0.7	0.017
C-reactive protein (mg/dL)	16.9 ± 13.7	14.3 ± 12.6	0.611*
Lactate level (mmol/L)	3.5 ± 3.2	6.3 ± 5.1	0.013*
SOFA score	3.7 ± 2.3	4.5 ± 1.7	0.004*
Mechanical ventilation	6 (6.9)	1 (5.9)	1.000**
Worst MAP (mmHg)	71.6 ± 15.3	67.6 ± 14.7	0.320
Vasopressor use	25 (28.7)	8 (47.1)	0.138
Time to surgery (h)	9.0 ± 5.6	6.6 ± 4.2	0.073*

Data are presented as mean ± SD, or number (%)

*WBC* white blood cell; *SOFA* sequential organ failure assessment; *MAP* mean arterial pressure

*Mann–Whitney’s U-test

**Fisher’s exact test

**Table 3:** Operation related factors

Characteristics	Survive to discharge (N = 87)	In-hospital mortality (N = 17)	*P-*value
Type			1.000
Elective	18 (20.9)	3 (17.6)	
Emergency	69 (79.1)	14 (84.4)	
Perforation site			0.294
Stomach	8 (9.2)	1 (5.9)	
Small intestine	32 (36.7)	10 (58.9)	
Appendix	2 (2.3)	0 (0.0)	
Large intestine	45 (51.7)	6 (35.3)	
Perforation cause			
Solid tumor	23 (26.4)	6 (35.3)	0.555
Lymphoma	2 (2.3)	4 (23.5)	0.006*
Ascites			0.036
Clear	28 (31.8)	1 (5.9)	
Contaminated	60 (68.2)	16 (94.1)	
Stoma formation	53(61.6)	10 (58.8)	0.828
Intraoperative input/output balance (mL)	1911.0 ± 1829.6	2107.0 ± 1584.6	0.435**
Intraoperative transfused RBC unit (n)	± 1.6	1.8 ± 3.3	0.242**

Data are presented as mean ± SD, or number (%)

*RBC* red blood cell

*Fisher’s exact test

**Mann–Whitney’s U-test

**Table 4:** Postoperative factors

Characteristics	Survive to discharge (N = 87)	In-hospital mortality (N = 17)	*P-* value
Postoperative lactate level (mmol/L)	3.6 ± 3.0	7.9 ± 4.9	< 0.001*
Postoperative lactate clearance (%)	− 30.7 ± 89.4	− 50.0 ± 63.8	0.096*
Perforation related re-operation	2 (3.2%)	1 (7.7%)	0.440

Data are presented as mean ± SD, or number (%)

*Mann–Whitney’s U-test

Multivariate analysis revealed that preoperative hemoglobin level (HR 0.707, 95% CI 0.520–0.959, *P* = 0.026), lymphoma as a cause of perforation (HR 8.852, 95% CI 1.262–62.087, *P* = 0.028), and postoperative lactate level (HR 1.259, 95% CI 1.084–1.463, *P* = 0.003) had significant effects on in-hospital mortality after surgery for GI perforation (Table 5). The model fitness was assessed by the Hosmer–Lemeshow test and was adequate (P = 0.264). Nagelkerke R^2^ was 0.425.

**Table 5:** Multivariable analysis of risk factors for in-hospital mortality

Characteristics	HR	95% CI	*P-*value
Preoperative hemoglobin (g/dL)	0.707	0.520–0.959	0.026
Lymphoma	8.852	1.262–62.087	0.028
Postoperative lactate level (mmol/L)	1.259	1.084–1.463	0.003

*HR* hazard ratio; *CI* confidence interval

**Fig. 2:**
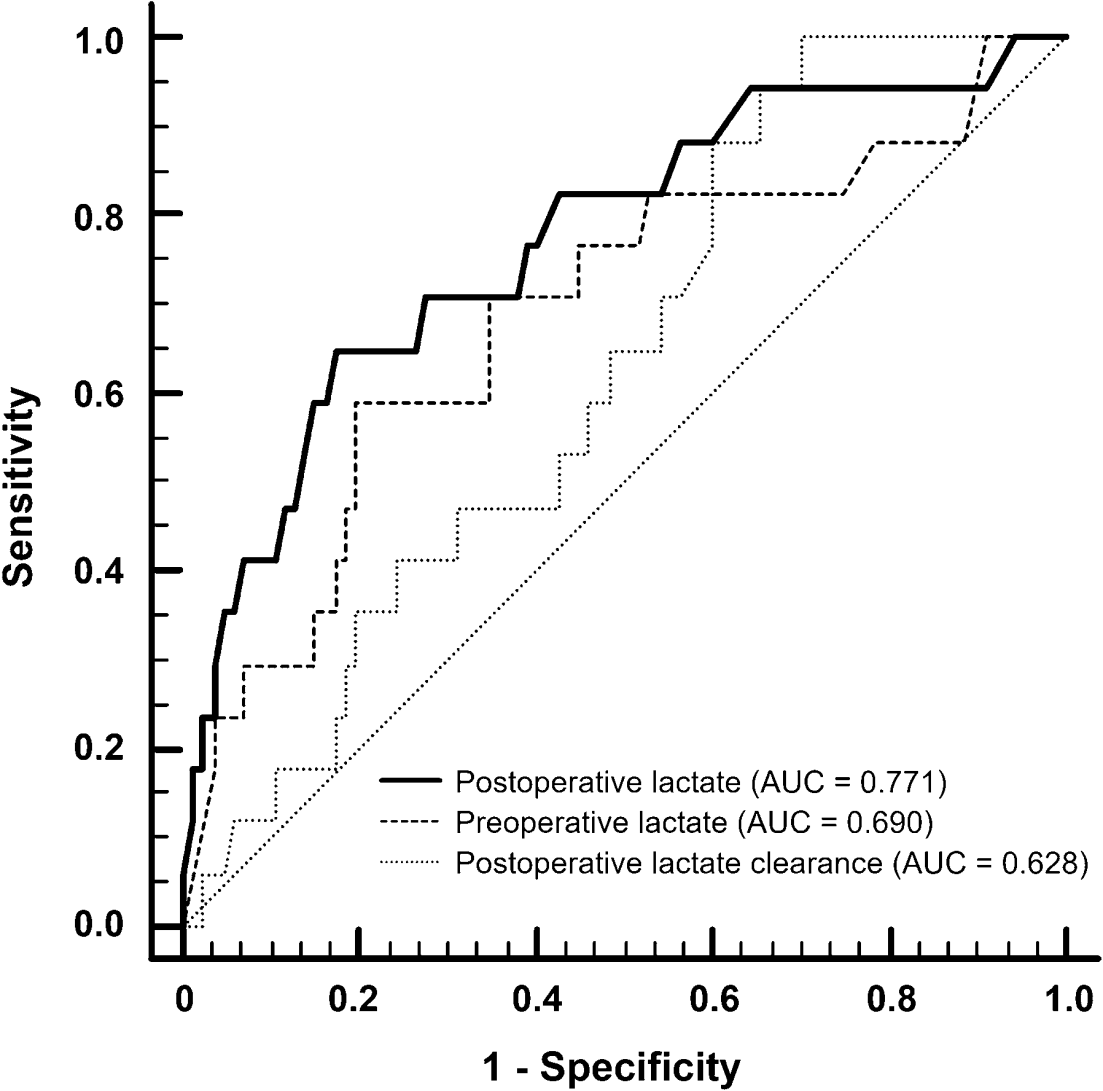
Receiver operating characteristic curves for prediction of in-hospital mortality after surgery for GI perforation according to pre and postoperative lactate levels and postoperative lactate clearance

In the ROC curve analysis, an AUC of 0.771 (95% CI 0.640–0.902) in the postoperative lactate was higher than those of preoperative lactate (AUC of 0.692, 95% CI 0.544–0.839) and postoperative lactate clearance (AUC of 0.628, 95% CI 0.528–0.721) (Fig. 2). When comparing ROC curves, the AUC of postoperative lactate was significantly higher than that of preoperative lactate (*P* = 0.019). Optimal cut-off values for pre and postoperative lactate levels and postoperative lactate clearance were 4.55, 5.95, and 15.33, respectively (Table 6 and Additional file 1: Table S1).

**Table 6:** Receiver operating characteristic curve analysis

Characteristics	AUC	95% CI	Cut-off	Sensitivity	Specificity	Positive predictive value	Negative predictive value	Positive likelihood ratio	Negative likelihood ratio
Preoperative lactate level (mmol/L)	0.690	0.592–0.777	4.5	58.82	80.46	37.0	90.9	3.01	0.51
Postperative lactate level (mmol/L)	0.771	0.678–0.848	5.9	64.7	82.8	42.3	92.3	3.75	0.43
Postoperative lactate clearance (%)	0.628	0.528–0.721	15.33	100.0	29.9	21.8	100.0	1.43	0.00

*AUC* area under curve; *CI* confidence interval

## Discussion

This study analyzed the association between pre and postoperative lactate and postoperative lactate clearance and in-hospital mortality. The results showed that postoperative lactate is the best predictor of in-hospital mortality. In addition, postoperative lactate was also identified as an independent risk factor for in-hospital mortality along with preoperative hemoglobin and lymphoma in the results of multivariate analysis performed with other variables.

Lactate is the final metabolite of anaerobic metabolism produced by decreased blood flow which leads to tissue-wide hypoxia. Septic shock is one of the common causes of hyperlactatemia or lactic acidosis. Lactate is an important biomarker to predict the prognosis of critically ill patients. Since lactate concentration varies according to the production and elimination of lactate in critically ill patients [18, 19], serial lactate measurement is recommended. However, the best time to measure lactate remains unclear because interpreting lactate concentration is challenging due to complex pathophysiology [20].

Studies of lactate levels at specific time points for the prediction of mortality in patients with GI perforation have been conducted steadily. Shimazaki et al. [4] studied postoperative lactate levels as a prognostic factor in patients with colorectal perforation, with the postoperative lactate level being higher in the mortality group than the survival group; while these findings were similar to those of this study with regards to the postoperative lactate levels, the reported levels of postoperative lactate were lower. This could be due to the inclusion of patients without shock. Other studies of perioperative risk factors for mortality after GI surgery indicated that pre and postoperative hyperlactemia were risk factors of mortality [2, 21]. In the study by Lee et al. [2], pre and postoperative lactate levels were not identified as risk factors for in-hospital mortality after emergency GI surgery. According to the results of the study by Jung et al. [21] when the lactate level alone is used, sensitivity and specificity for mortality prediction are insufficient. However, when the lactate level is used with quick SOFA score, sensitivity and specificity increased up to 72%.

As repeated lactate measurement gained interests, the concept of lactate clearance emerged [22, 23]. Lactate clearance, is also known as a useful biomarker for predicting mortality in critically ill patients [24]. However, since the patient’s response to the treatment during the medical treatment such as fluid resuscitation or intravenous antibiotics and during the surgical treatment are completely different, the lactate clearance cannot be interpreted in the same way. In patients with GI perforation, peritonitis and septic shock rapidly progress due to the significant number of Gram-negative bacilli from bowel spillage, which in turn causes the release of inflammatory cytokines such as interleukin-1 and interleukin-6 [4]. This response heightens as the bacteria load increases over time, but the clinical course improves rapidly after the cause of septic shock is eliminated through the surgery [1]. For this reason, postoperative lactate clearance is used [25] instead of lactate clearance during the clinical course.

In this study, we thought that postoperative lactate clearance would be a better predictor of in-hospital mortality than lactate concentration at a single point because postoperative lactate clearance reflects all the effects of the interval from diagnosis to surgery, intraoperative resuscitation, stress caused by the invasive surgical process, and operative findings. However, the strongest predictors of in-hospital mortality was the postoperative lactate, not the postoperative lactate clearance. Possible explanation for this result can be characteristics of injured organs [14]. During surgery for GI perforation, a large amount of fluid is inevitably lost. If too much fluid resuscitation is performed to compensate this, the operation may proceed in a different direction due to bowel edema. Therefore, careful fluid resuscitation is required, which may have resulted in decreased performance of postoperative lactate clearance.

Other known risk factors of mortality in GI perforation include a high SOFA score, low preoperative WBC count (< 4000/μL), preoperative anemia, preoperative hypoalbuminemia, colorectal perforation, cancer related perforation and delayed surgery [2, 4, 5]. Although only preoperative anemia demonstrated a statistically significant association in this study, the trend for the majority of the known risk factors was consistent with previous studies. The superior prognosis of colorectal perforation in this study may be due to the inclusion of relatively ‘clean’ perforations that occurred during colonoscopy. Time to surgery was shorter in the mortality group, but not with statistical significance. This is probably because the means of both groups was 6.9 ± 4.6 and 8.7 ± 5.4, respectively, which was shorter than the cut-off points shown in previous studies of 12–24 h [4, 26].

Despite obtaining meaningful results, there are several limitations to this study. First, the sample size was not significant enough to justify the conclusions. Since the study population was limited only to patients who required intensive care unit (ICU) admission after surgery, the sample size was inevitably small. However, the severity of patients’ disease was higher, and characteristics were more homogenous than previous studies. Therefore, the results of this study can be used as a basis for establishing a hypothesis and designing a prospective study. Second, patients who did not admitted ICU after surgery were excluded due to the absence of measured preoperative lactate levels, leading to selection bias and thereby affecting the characteristics of the survival group. This selection bias may be the reason for the insufficient significance of the SOFA score in the multivariate analysis. Third, data on operative fields such as ascites characteristics may be subjective because they were retrospectively collected using surgical records generated by numerous surgeons. Fourth, as postoperative and delta lactate levels can only be calculated after the operation, patients with low surgical benefit could not be screened in advance.

## Conclusions

In summary, of pre and postoperative lactate and postoperative lactate clearance of patients who underwent surgery for GI perforation, postoperative lactate was the strongest predictor for in-hospital mortality.

## Supplementary Information

**Additional file 1: Table S1.** Receiver operating characteristic curve analysis.

## Data Availability

Data of the current study are available from the corresponding author on reasonable request.

## Abbreviations

AUC: Area under the curve
CCI: Charlson Comorbidity Index
CRP: C-reactive protein
GI: Gastrointestinal
MPI: Mannheim Prognostic Index
POSSUM: Physiological and Operative Severity Score for enUmeration of Mortality and morbidity
ROC: Receiver operating characteristic
SNUH: Seoul National University Hospital
SOFA: Sequential Organ Failure Assessment
WBC: White blood cell
