# Supplementary material for: Pre and postoperative lactate levels and lactate clearance in predicting in-hospital mortality after surgery for gastrointestinal perforation
Source: BMC Surg. 2022 Mar 9;22:93. doi: 10.1186/s12893-022-01479-1 (PMC8908642; doi:10.1186/s12893-022-01479-1)
Supplement: Supplementary file 1 — Additional file 1: Table S1. Receiver operating characteristic curve analysis. [file 12893_2022_1479_MOESM1_ESM.docx]

**Supplementary Table 1. Receiver operating characteristic curve analysis**

| Characteristics | Sensitivity | 95% CI | Specificity | 95% CI | Positive predictive value | 95% CI | Negative predictive value | 95% CI | Positive likelihood ratio | 95% CI | Negative likelihood ratio | 95% CI |
| --- | --- | --- | --- | --- | --- | --- | --- | --- | --- | --- | --- | --- |
| Preoperative lactate level (mmol/L) | 58.82 | 32.9-81.6 | 80.6 | 70.6-88.2 | 37.0 | 24.7-51.3 | 90.9 | 84.9-94.7 | 3.01 | 1.68-5.39 | 0.51 | 0.29-0.91 |
| Postperative lactate level (mmol/L) | 64.7 | 38.3-85.8 | 82.76 | 73.2-90.0 | 42.3 | 29.1-56.7 | 92.3 | 86.2-95.8 | 3.75 | 2.10-6.70 | 0.43 | 0.22-0.82 |
| Postoperative lactate clearance (%) | 100.0 | 80.5-100.0 | 29.89 | 20.5-40.6 | 21.8 | 195.0-24.2 | 100.0 | N/A | 1.43 | 1.24-1.64 | 0.00 | N/A |

CI, confidence interval.
